# Genomic analysis of extended-spectrum β-lactamase-producing *Escherichia coli* and *Klebsiella pneumoniae* isolated from a dog with a severe episode of self-trauma and urinary tract infection

**DOI:** 10.1007/s11259-026-11615-4

**Published:** 2026-11-06

**Authors:** Camile Pereira Repossi, Sara Colodetti Paiva, Matheus Nunes Simões, Bruna Modenese Fernandes, João Vitor Oliveira Silva, Thiago Aragão de Araújo, Carolina Magri Ferraz, Igor Luiz Salardani Senhorello, Gabriel Augusto Marques Rossi, João Pedro Rueda Furlan

**Affiliations:** 1 Department of Veterinary Medicine, University Vila Velha (UVV), Av. Comissário José Dantas de Melo, n.21, Vila Velha, ES 29102-920 Brazil; 2 Department of Animal Clinic, Surgery and Reproduction, School of Veterinary Medicine, São Paulo State University (UNESP), 793 Clóvis Pestana St, Araçatuba, São Paulo 16050-680 Brazil; 3 Department of Pharmaceutical Sciences, Health Sciences Center, Federal University of Paraíba, João Pessoa, Paraíba Brazil

**Keywords:** Antimicrobial resistance, *Enterobacterales*, β-lactamase, Canine, Veterinary medicine

## Abstract

Extended-spectrum β-lactamase (ESBL)-producing *Enterobacterales* are emerging pathogens of major importance in veterinary medicine. This study describes the clinical, microbiological, and genomic characteristics of ESBL-producing *Escherichia coli* and *Klebsiella pneumoniae* isolated from a dog with paraparesis and concurrent pyelonephritis, chronic cystitis, and a self-traumatized limb lesion. A 12-year-old mixed-breed female dog underwent repeated antimicrobial therapy during hospitalization. Urine obtained by cystocentesis and wound samples were cultured, and ESBL-producing strains were characterized by antimicrobial susceptibility testing and whole-genome sequence-based analysis. CTX-M-55-producing *E. coli* sequence type (ST) 457, phylogroup F, O11:H25, and *fimH*145, was recovered from urine and wound samples, while the wound sample also yielded a CTX-M-15- and SHV-2-producing *K. pneumoniae* ST39, KL149:OL2α.2. The two closely related *E. coli* strains differed by 96 single nucleotide polymorphisms, despite sharing the same ST, resistome, plasmid replicons, and virulence profile. ESBL-positive strains carried multiple virulence genes, with the *E. coli* strains classified as extraintestinal pathogenic *E. coli* strains. To the best of our knowledge, this is the first report of ESBL-producing *K. pneumoniae* ST39 in an animal in Brazil. The co-occurrence of ESBL-producing *E. coli* ST457 and *K. pneumoniae* ST39 in the same dog highlights the potential for companion animals to harbor clinically relevant multidrug-resistant lineages and reinforces the need for continuous antimicrobial resistance monitoring and antimicrobial stewardship in this sector.

**Supplementary Information:**

The online version contains supplementary material available at https://doi.org/10.1007/s11259-026-11615-4.

## Background

Antimicrobial resistance (AMR) is an increasing concern in companion animal medicine, particularly because of the emergence and dissemination of multidrug-resistant (MDR) bacteria. Among them, members of the order *Enterobacterales* are important causes of canine infections and may contribute to the circulation of antimicrobial-resistant bacteria and antimicrobial resistance genes (ARGs) within households and veterinary healthcare settings, highlighting their relevance from a One Health perspective (Haenni et al. 2022; Menezes et al. 2024). Of particular concern is the production of extended-spectrum β-lactamases (ESBLs), which hydrolyze extended-spectrum cephalosporins and aztreonam, thereby substantially limiting therapeutic options (Yassara et al. 2025).

*Escherichia coli* and *Klebsiella pneumoniae* are among the most clinically relevant *Enterobacterales* associated with extraintestinal infections in dogs, particularly urinary tract infections. ESBL-producing strains of both species have been increasingly reported among canine clinical strains, and bacterial cystitis represents one of the most frequent clinical manifestations associated with these organisms (Woerde et al. 2023; Facchin et al. 2025). In addition to community-associated infections, veterinary hospitalization may facilitate the acquisition, persistence, and dissemination of ESBL-producing *Enterobacterales*, contributing to the occurrence of healthcare-associated infections (Woerde et al. 2023; Silva et al. 2024).

Whole-genome sequencing (WGS) enables comprehensive characterization of AMR determinants and high-resolution assessment of genomic relatedness among bacterial strains (Leal-Vélez et al. 2025). In the context of infections involving multiple anatomical sites, this approach can be particularly valuable for determining whether strains recovered from different clinical specimens represent the same bacterial strain and for characterizing the coexistence of distinct resistant pathogens within an individual host (Haenni et al. 2022).

Here, we describe the clinical, phenotypic, and genomic characterization of *E. coli* and *K. pneumoniae* strains producing ESBLs recovered from a paraparetic dog presenting with a urinary tract infection and an infected limb lesion due to self-trauma. The concurrent detection of two distinct ESBL-producing *Enterobacterales*, together with the recovery of closely related *E. coli* strains at different anatomical sites, illustrates the diagnostic, therapeutic, and infection-control challenges posed by MDR bacterial infections in companion animals.

## Case report

A 12-year-old mixed-breed female dog weighing 27.35 kg developed sudden-onset non-ambulatory paraparesis in July 2025. At that time, the owners did not pursue a definitive diagnosis, and the condition was presumptively attributed to hip dysplasia.

During November 2025, four months after the onset of paraparesis, the dog was hospitalized for the first time because of anorexia, lethargy, hematuria, and myiasis affecting the pelvic limbs secondary to acute moist dermatitis associated with impaired mobility. During the four-day hospitalization, empirical intravenous antimicrobial therapy with ceftriaxone (50 mg/kg, IV, q12h) and metronidazole (15 mg/kg, IV, q12h) was administered. The dog was subsequently discharged and prescribed amoxicillin–clavulanate (25 mg/kg, PO, q12h) for 10 days.

**Fig. 1:**
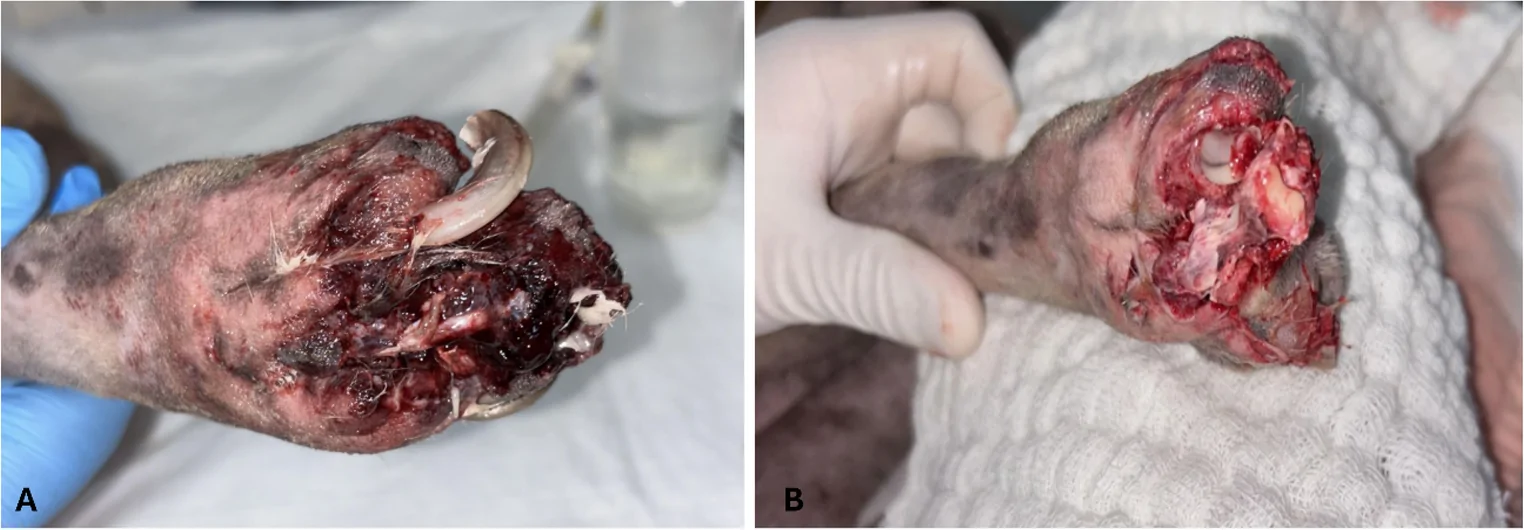
Lesion in the distal region of the left pelvic limb of the 12-year-old female dog. (**A**) Lesion on the day of admission, showing an extensive, contaminated wound with areas of necrosis. (**B**) Wound following surgical debridement

Five months after the first hospitalization, during April 2026, the dog was admitted as an emergency following a severe episode of self-trauma. Physical examinations revealed lethargy, hyperthermia (39.4 °C), and approximately 5% dehydration. A large, contaminated lesion with phalangeal exposure was observed on the left pelvic limb (Fig. 1A). Marked pyuria and episodes of fetid, dark-colored diarrhea were also noted. Based on the clinical findings and suspicion of sepsis and pyelonephritis, supportive therapy was initiated with lactated Ringer’s solution (3 mL/kg/h, IV), multimodal analgesia, and empirical intravenous antimicrobial therapy with ceftriaxone (30 mg/kg, IV, q12h), enrofloxacin (10 mg/kg, IV, q12h), and metronidazole (15 mg/kg, IV, q12h).

On the day of admission, the patient underwent surgical debridement of the limb lesion (Fig. 1B). Immediately afterward, wound management was initiated by cleansing the lesion with sterile gauze soaked in saline solution, followed by application of Vetaglós healing ointment (Vetnil Indústria Veterinária Ltda., Brazil) and a calcium alginate dressing. The dressing was secured with bandages and elastic wrap and replaced every 24 h. An Elizabethan collar was maintained throughout the treatment period to prevent further self-trauma and preserve dressing integrity.

Concurrently, samples were collected for microbiological investigation. Urine was obtained by cystocentesis, and wound exudate was collected using a sterile swab; both samples were submitted for bacterial culture and antimicrobial susceptibility testing. Venous blood was also collected for hematological and biochemical evaluation. Hematological analysis revealed leukocytosis (17,700/µL) with neutrophilia (15,930/µL) without a left shift. Biochemical analysis showed elevated blood urea nitrogen (71.2 mg/dL), whereas serum creatinine remained within the reference range (0.98 mg/dL). Mild hypoalbuminemia (2.5 g/dL) was also detected.

Abdominal ultrasonography revealed bilateral nephropathy. The right kidney exhibited moderate hydronephrosis secondary to an obstructive process associated with right ureterolithiasis, whereas the left kidney showed mild pyelectasis; the appearance of the renal pelvic contents in both kidneys was suggestive of pyelonephritis. The urinary bladder showed findings consistent with chronic cystitis, including urinary sediment, microcalculi, and a small amount of intravesical gas, raising suspicion of early-stage emphysematous cystitis.

The urine and wound swab samples were processed at a clinical microbiology laboratory. The urine sample was inoculated onto Cystine-Lactose-Electrolyte-Deficient agar (HiMedia, India), whereas the wound swab sample was inoculated onto Blood Agar Base (HiMedia, India) supplemented with 5% sheep blood and MacConkey agar (Kasvi, Spain). The plates were incubated aerobically at 35 ± 2 °C for 24 h. Following bacterial growth, colonies were evaluated according to their morphological characteristics, and smears were prepared for Gram staining.

Microscopic examination identified four bacterial strains, including one Gram-positive coccus arranged in grape-like clusters from the wound swab and three Gram-negative bacilli, one from the urine sample and two from the wound swab. The Gram-positive coccal strain was subjected to a catalase test using 3% hydrogen peroxide (Rioquímica, Brazil) and a coagulase test using rabbit plasma (Newprov, Brazil). The three Gram-negative bacillary strains were subjected to oxidase testing and biochemical identification using the Bactray 1 and Bactray 2 systems, following the manufacturer’s recommendations for preparation, incubation conditions, and result interpretation (LaborClin, Brazil). Accordingly, an *E. coli* strain (HV-ENT-001) was identified from the urine sample, whereas *E. coli* (HV-ENT-002), *K. pneumoniae* (HV-ENT-003), and *Staphylococcus aureus* (HV-ENT-004) were isolated from the limb lesion.

Antimicrobial susceptibility testing (AST) was performed using the disk diffusion method. The antimicrobial disks used were selected based on the requests of the attending veterinarians as part of the initial clinical management of the patient, before bacterial identification. For *Enterobacterales*, the antimicrobial panel comprised amoxicillin-clavulanic acid (AMC, 20/10 µg), cefadroxil (CFR, 30 µg), cephalexin (CFE, 30 µg), ceftriaxone (CRO, 30 µg), chloramphenicol (CHL, 30 µg), doxycycline (DOX, 30 µg), enrofloxacin (ENR, 5 µg), gentamicin (GEN, 10 µg), nitrofurantoin (NIT, 300 µg), and trimethoprim-sulfamethoxazole (SXT, 1.25/23.75 µg). ENR and NIT were not tested against the *K. pneumoniae* strain because interpretive criteria for these antimicrobial-species combinations are not provided in the adopted guidelines. For the *S. aureus* strain, the tested antimicrobials were ENR, SXT, AMC, and CFE. AST results were preferentially interpreted according to veterinary-specific breakpoints established by the Clinical and Laboratory Standards Institute (CLSI, 2024). Because veterinary-specific CLSI VET01S™ breakpoints were unavailable for ceftriaxone (CRO), cephalexin (CFE), and cefadroxil (CFR), interpretive criteria established by the European Committee on Antimicrobial Susceptibility Testing (EUCAST, 2026) were applied to these antimicrobials. CLSI VET01S™ criteria were applied to the remaining antimicrobial-species combinations. *E. coli* ATCC^®^ 25,922 and *S. aureus* ATCC^®^ 25,923 were included as quality-control strains.

*E. coli* strains (HV-ENT-001 and HV-ENT-002) presented the same susceptibility profiles. Both strains were resistant to AMC, CFR, CFE, CRO, ENR, GEN, DOX, and SXT, but remained susceptible to NIT and CHL. The *K. pneumoniae* strain HV-ENT-003 exhibited the same susceptibility profile as the *E. coli* strains for the antimicrobials tested; ENR and NIT were not evaluated because interpretive criteria for *K. pneumoniae* were unavailable in the adopted guidelines. The *S. aureus* strain (HV-ENT-004) was resistant to ENR and SXT but susceptible to AMC and CFE (Table 1).

**Table 1:** Antimicrobial susceptibility of strains from this study

Strain	Species	Antimicrobials^1^									
		AMC	CFR	CFE	CRO	CHL	DOX	ENR	GEN	SXT	NIT
HV-ENT-001	*E. coli*	R	R	R	R	S	R	R	R	R	S
HV-ENT-002	*E. coli*	R	R	R	R	S	R	R	R	R	S
HV-ENT-003	*K. pneumoniae*	R	R	R	R	S	R	-	R	R	-
HV-ENT-004	*S. aureus*	S	-	S	-	-	-	R	-	R	-

^1^*R* resistant, *S* susceptible, - not tested

Notably, *E. coli* and *K. pneumoniae* strains presented multidrug resistance profiles following the criteria of Magiorakos et al. (2012). Moreover, these strains belonged to pathogen-resistance combinations classified by WHO as critical priority, namely third-generation cephalosporin-resistant *E. coli* and *K. pneumoniae* (Sati et al. 2025). Based on their AMR profiles, these strains were further evaluated for phenotypic ESBL production using the double-disk synergy test (Drieux et al. 2008). Bacterial suspensions were adjusted to a 0.5 McFarland standard and evenly inoculated onto Mueller-Hinton agar plates (Kasvi, Spain). An AMC disk (20/10 µg) was placed in the center of each plate, with aztreonam (30 µg), cefepime (30 µg), ceftazidime (30 µg), and CRO (30 µg) disks positioned 20 mm from the amoxicillin–clavulanic acid disk, measured center-to-center. Plates were incubated aerobically at 35 ± 2 °C for 18–24 h. ESBL production was considered positive when enhancement of the inhibition zone of any β-lactam disk toward the AMC disk was observed, indicating synergy with clavulanate. The test showed a positive synergy effect, providing phenotypic evidence consistent with ESBL production.

During hospitalization, urinary management represented the main technical challenge. Recurrent urethral catheter obstruction occurred due to the accumulation of purulent material and debris associated with the severe urinary tract infection involving both the upper and lower urinary tracts. These episodes required frequent bladder flushing with saline every 2 h to maintain catheter patency and urinary flow. Although the patient was urgently referred for specialist nephrological evaluation, the owners elected to discharge the dog against medical advice three days after admission. No subsequent follow-up was obtained, and contact with the patient was lost, precluding assessment of the final clinical outcome.

To further characterize the ESBL-producing *Enterobacterales* involved in this case, the strains HV-ENT-001, HV-ENT-002, and HV-ENT-003 were subjected to whole-genome sequence-based analysis. Accordingly, genomic DNAs were extracted using the DNeasy Blood & Tissue Kit (QIAGEN, Germany) and were sequenced using the Illumina MiSeq i100 platform (Illumina Inc., USA). Genomes were *de novo* assembled by SPAdes v.4.3.0 (https://github.com/ablab/spades) and quality-checked using CheckM v.1.2.2 (https://github.com/ecogenomics/checkm) (Supplementary Table S1). Antimicrobial resistance, virulence, plasmid replicons, and typing were determined using tools available at Pathogenwatch (https://pathogen.watch/) and Center for Genomic Epidemiology (https://genepi.dk/). *E. coli* phylogenetic group was analyzed using ClermonTyping v.23.06 (https://github.com/iame-researchCenter/ClermonTyping). Genomic relatedness between the two *E. coli* strains was further assessed by single nucleotide polymorphisms (SNP)-based analysis using CSI Phylogeny v.1.4 (Kaas et al. 2014). The genomic location of ESBL genes was predicted using mlplasmids (Arredondo-Alonso et al. 2018), and the corresponding contigs were further examined using Nucleotide BLAST (https://blast.ncbi.nlm.nih.gov/Blast.cgi). Association with a specific plasmid replicon was inferred only when the ESBL gene and replicon were located on the same contig. These associations were considered in silico predictions due to the limitations of short-read sequencing. All tools were used with default parameters.

Interestingly, *E. coli* strains HV-ENT-001 (urine) and HV-ENT-002 (wound swab) presented highly similar genomic profiles. Both strains were identified as belonging to sequence type (ST) 457, phylogroup F, serotype O11:H25, and *fimH*145. Resistome comprised *bla*_CTX−M−55_, *bla*_TEM−1B_, *aph(6)-Id*, *aph(3’’)-Ib*, *aph(3’)-Ia*, *tet(A)*, *sul2*, and *dfrA14*, in addition to quinolone resistance-associated mutations in GyrA (S83L and D87Y), ParC (S80I), and ParE (S458A), corroborating the AMR phenotype-genotype relationship. Plasmid replicons IncFII(pHN7A8), p0111, IncI1-I(Alpha), IncI2, IncFIB(AP001918), and IncFIC(FII), were identified. Virulome results included *aslA*,* air*,* anr*,* cba*,* cea*,* chuA*,* cia*,* cma*,* csgA*,* cvaC*,* eilA*,* espY2*,* fdeC*,* gad*,* hha*,* hlyE*,* hlyF*,* hra*,* iss*,* iucC*,* iutA*,* kpsE*,* kpsMII*,* lpfA*,* nlpI*,* ompT*,* papA_F1651A*,* papC*,* shiA*,* shiB*,* sitA*,* terC*,* tir*,* traJ*,* traT*,* yehA*,* yehB*,* yehC*,* yehD*,* yfcV*, and *yghJ.* Both strains were classified as extraintestinal pathogenic *E. coli* (ExPEC) according to the molecular criteria proposed by Johnson et al. (2003), which require the presence of at least two of five virulence markers (*pap*, *sfa*/*foc*, *afa*/*dra*, *kpsMT II*, and *iutA*). Accordingly, both strains fulfilled this criterion by carrying *papA*, *papC*, *kpsMII*, and *iutA*. SNP-based analysis revealed 96 SNP differences between these strains. Therefore, considering their overall genomic profiles, they were regarded as closely related strains belonging to the same ST457 lineage.

*K. pneumoniae* strain HV-ENT-003 (wound swab) belonged to ST39 and serotype KL149:OL2α.2. This strain harbored both *bla*_CTX−M−15_ and *bla*_SHV−2_, together with an extensive resistome comprising *bla*_OXA−1_, *bla*_TEM−1B_, *aac(6’)-Ib-cr*, *qnrB19*, *oqxA*, *oqxB*, *aph(6)-Id*, *aph(3’’)-Ib*, *aac(3)-IIa*, *sul2*, *dfrA14*, *catB3Δ*, and *fosA*^KP^. Plasmid replicons IncFII(K), IncFIB(K), IncX1, Col(pHAD28), and ColpVC, and virulence genes *mrkA*,* mrkB*,* mrkC*,* mrkD*,* mrkF*,* mrkH*,* mrkI*, and *mrkJ* were also found.

The *bla*_CTX−M−55_ and *bla*_CTX−M−15_ genes were predicted to be located on plasmid contigs and were flanked by IS*Ecp1* and *wbuC*. In *E. coli* strains, the *bla*_CTX−M−55_-containing contigs also harbored IncFIB(AP001918)/IncFIC(FII) replicon sequences and virulence-associated genes, including *iutA*, *iucC*, *hlyF*, *ompT*, *sitA*, *traT*, *cma*, *traJ*, *anr*, *cvaC*, and *cba*. In the *K. pneumoniae* strain, *bla*_CTX−M−15_ was embedded within a Tn*3*-related AMR module containing *bla*_CTX−M−15_, *bla*_TEM−1B_, *aph(6)-Id*, *aph(3’’)-Ib*, and *sul2*, although no plasmid replicon was detected on the same contig. On the other hand, *bla*_SHV−2_ was predicted to be chromosomally located, with its surrounding region lacking any mobile genetic elements.

## Discussion & conclusion

In this case report, the dog developed sudden-onset paraparesis without a definitive etiological diagnosis. However, an acute thoracolumbar or lumbosacral spinal cord lesion was considered a possible underlying cause, as intervertebral disc disease and aortic thromboembolism are among the most common causes of acute paralysis in dogs presenting to emergency clinics (Rossi et al. 2020). Spinal cord injury, particularly at the suprasacral level, can result in neurogenic lower urinary tract dysfunction, including impaired bladder emptying and urinary retention, thereby predisposing affected animals to urinary tract infections (Hu et al. 2016). In the present clinical case, this neurological dysfunction may have contributed to the development of urinary tract infection, as evidenced by the concomitant occurrence of cystitis and renal involvement.

The self-mutilation of the affected limb observed in this dog may also have been associated with the neurological dysfunction that developed following the sudden-onset paraparesis. Neurological lesions affecting the spinal cord may alter sensory processing and nociception, potentially resulting in neuropathic pain, paresthesia, or dysesthesia, which can manifest clinically as excessive licking, biting, and self-mutilation (Moore 2016). Similar associations between neurological dysfunction, altered sensory function, and self-traumatic behavior have been described in dogs with sensory and autonomic neuropathies, in which self-mutilation of the extremities occurred concurrently with proprioceptive and urinary dysfunction (Leonard et al. 2022). In this case, the temporal association between the onset of paraparesis and subsequent limb self-mutilation raises the possibility that abnormal sensory input secondary to neurological impairment contributed to the development of the limb lesion. Moreover, the resulting self-trauma may have disrupted the skin barrier and facilitated secondary bacterial infection, potentially explaining the polymicrobial infection identified in the lesion. Concurrent urinary abnormalities, including chronic cystitis, ureterolithiasis, and hydronephrosis, may likewise have been influenced by neurological dysfunction and impaired lower urinary tract function. Although a direct causal relationship cannot be established, the coexistence of neurological impairment, urinary tract disease, and self-inflicted limb injury suggests that these conditions may have been interconnected in the clinical progression of this patient.

The polymicrobial nature of the wound, involving multiple bacterial species, further suggests a complex secondary infection following disruption of the cutaneous barrier, although the individual contribution of each microorganism cannot be determined. Notably, closely related *E. coli* strains belonging to the same ST457 lineage were identified in urine collected by cystocentesis and in the swab obtained from the limb lesion. However, the 96-SNP distance cannot alone support direct within-host dissemination, as SNP-based relatedness in *E. coli* is highly context-dependent (Nesporova et al. 2020; Watt et al. 2025). The presence of these strains in the limb lesion may reflect a true infectious process, especially considering their classification as ExPEC and the fact that they harbor multiple virulence-associated genes involved in adhesion and tissue colonization, biofilm formation, iron acquisition, and evasion of host immune responses (Subashchandrabose and Mobley 2015; Sora et al. 2021). Alternatively, the recovery of *E. coli* from the limb lesion may have resulted from direct or indirect contamination by urine, potentially facilitated by biting of the self-traumatized area and subsequent contact with urine-contaminated oral surfaces. Therefore, although the genetic similarity between *E. coli* strains suggests a common source, the available data do not allow us to determine whether *E. coli* actively contributed to the limb infection or was secondarily introduced through urinary contamination and self-trauma.

*E. coli* ST457 has previously been identified among ESBL-producing strains from companion animals, being the most frequent clone among ESBL-producing strains recovered from urine samples from dogs and cats in a veterinary teaching hospital in Taiwan (Huang et al. 2020). In Brazil, ST457 has also been identified among the predominant extended-spectrum cephalosporin-resistant *E. coli* clones in companion animals, although the ESBLs detected were mainly CTX-M-2 and CTX-M-8 rather than CTX-M-55 (Melo et al. 2018). Notably, CTX-M-55-producing ST457 *E. coli* has been reported among ExPEC from poultry in Brazil, carrying *bla*_TEM−1B_, as observed in our strain, together with multiple additional AMR and virulence determinants (Cunha et al. 2017). The potential for zoonotic dissemination of this lineage is further supported by its identification in humans and companion animals, including a CTX-M-55-producing strain shared between a dog and its owner in Portugal (Menezes et al. 2024). Furthermore, CTX-M-55-producing *E. coli* has been reported in human urinary tract infections and commercial meat, including chicken and pork, highlighting the circulation of this ESBL variant across human and food-associated reservoirs (Soncini et al. 2022).

*K. pneumoniae* ST39 has been reported in humans and companion animals. Notably, this lineage appears to be rare in Brazil, having been reported only once among human strains from transplanted patients (Raro et al. 2020). To the best of our knowledge, this is the first report of ESBL-producing *K. pneumoniae* ST39 in an animal in Brazil. Although ST39 has previously been detected in clinically ill companion animals in South Korea (Lee et al. 2021), its occurrence in veterinary strains and association with specific infections in dogs remain poorly documented. In the present case report, the presence of the complete *mrkABCDFHIJ* gene cluster, encoding the type 3 fimbrial system, may have favored adhesion, biofilm formation, and persistence of *K. pneumoniae* in the damaged tissue, as type 3 fimbriae are associated with adherence to extracellular matrix and biofilm formation (Paczosa and Mecsas 2016). However, biofilm-forming ability was not phenotypically evaluated, precluding confirmation of the functional contribution of these virulence-associated genes. Moreover, because *K. pneumoniae* was recovered from a polymicrobial wound, its specific contribution to the infectious process cannot be established, although ESBL-producing *K. pneumoniae* has been associated with both urinary tract and soft tissue infections in companion animals, with soft tissue infections representing the most frequent source of ESBL-positive strains in one large veterinary study (Ewers et al. 2014).

The strain harbored two ESBLs. CTX-M-15, which is among the predominant ESBL variants reported worldwide, and SHV-2, which is an older but unusual variant (Bassiouny et al. 2026; Paz et al. 2026). Despite the detection of *catB3*, the *K. pneumoniae* strain remained susceptible to chloramphenicol. Further sequence analysis revealed the presence of the truncated *catB3Δ*^443–633^ variant, which has been shown to be non-functional and unable to confer chloramphenicol resistance (Graf et al. 2024), explaining the observed genotype-phenotype discordance.

Repeated exposure to broad-spectrum antimicrobials throughout the clinical course may have exerted selective pressure favoring the selection or persistence of multidrug-resistant and ESBL-producing bacteria. Previous antibiotic exposure has been consistently associated with subsequent isolation or acquisition of antimicrobial-resistant bacteria, although the magnitude and duration of this effect vary according to the antimicrobial class and bacterial species (Sulis et al. 2022). Although a direct causal relationship between antimicrobial exposure and the emergence or persistence of the resistant strains identified in this patient cannot be established, repeated antimicrobial treatment may have contributed to their persistence and potentially limited subsequent therapeutic options. The absence of AST during previous episodes also highlights the importance of microbiological culture and susceptibility testing to support antimicrobial selection, particularly in recurrent or complicated infections. The ESBL-producing strains were recovered during empirical ceftriaxone therapy, but AST-guided treatment could not be implemented because the results became available only after discharge.

In summary, this case illustrates the complex interplay between neurological dysfunction, urinary tract disease, self-trauma, and secondary polymicrobial infection in a dog carrying clinically relevant ESBL-producing *E. coli* and *K. pneumoniae* lineages. The identification of CTX-M-55-producing *E. coli* ST457 and CTX-M-15- and SHV-2-producing *K. pneumoniae* ST39 in the same animal provides additional evidence that companion animals may harbor antimicrobial-resistant lineages of clinical relevance to veterinary and human health, consistent with previous reports (Menezes et al. 2024; Lee et al. 2021). These findings further emphasize the importance of AMR surveillance and antimicrobial stewardship in veterinary medicine, particularly the judicious use of broad-spectrum antimicrobials, to reduce the selection and persistence of MDR bacteria. From a One Health perspective, responsible antimicrobial use in companion animals is particularly relevant given their potential to harbor clinically important multidrug-resistant lineages.

## Supplementary Information

Below is the link to the electronic supplementary material.

Supplementary Material 1 (DOCX 29.2 KB)

## Data Availability

Whole-genome sequencing data generated in this study have been deposited in DDBJ/ENA/GenBank under BioProject accession number PRJNA1524134, with genome assembly accession numbers JCDQAR010000000 (HV-ENT-001), JCDQAQ010000000 (HV-ENT-002), and JCDQAP010000000 (HV-ENT-003).
