# Supplementary material for: Genomic analysis of extended-spectrum β-lactamase-producing Escherichia coli and Klebsiella pneumoniae isolated from a dog with a severe episode of self-trauma and urinary tract infection
Source: Vet Res Commun. 2026 Nov 6;51(1):35. doi: 10.1007/s11259-026-11615-4 (PMC13739995; doi:10.1007/s11259-026-11615-4)
Supplement: Supplementary file 1 [file 11259_2026_11615_MOESM1_ESM.docx]

**Supplementary Material**

**Genomic analysis of extended-spectrum β-lactamase-producing *Escherichia* *coli* and *Klebsiella* *pneumoniae* isolated from a dog with a severe episode of self-trauma and urinary tract infection**

**Authors:**

Camile Pereira Repossi^a^, Sara Colodetti Paiva^a^, Matheus Nunes Simões^a,^ Bruna Modenese Fernandes^a^, João Vitor Oliveira Silva^a^, Thiago Aragão de Araújo^a^, Carolina Magri Ferraz^a^, Igor Luiz Salardani Senhorello^b^, Gabriel Augusto Marques Rossi^a,*^, João Pedro Rueda Furlan^c,*^

**Affiliations:**

^a^ Department of Veterinary Medicine, University Vila Velha (UVV), Av. Comissário José Dantas de Melo, n.21, Vila Velha 29102-920, ES, Brazil.

^b^ Department of Animal Clinic, Surgery and Reproduction, São Paulo State University (UNESP), School of Veterinary Medicine, 793 Clóvis Pestana St., Araçatuba, São Paulo 16050-680, Brazil.

^c^ Department of Pharmaceutical Sciences, Health Sciences Center, Federal University of Paraíba, João Pessoa, Paraíba, Brazil.

*** Corresponding authors:**

Prof. Gabriel Augusto Marques Rossi (<gabriel.rossi@uvv.br>)

Prof. João Pedro Rueda Furlan (<joao.furlan@academico.ufpb.br>)

**Supplementary Table S1.** Genome assembly statistics of strains in this study

| **Strain** | **Species** | **Genome size (bp)^1^** | **Number of contigs** | **N50 value** | **Genome**  **coverage (×)** | **Completeness (%)** | **Contamination (%)** |
| --- | --- | --- | --- | --- | --- | --- | --- |
| HV-ENT-001 | *Escherichia coli* | 5318795 | 196 | 125522 | 57 | 99.97 | 0.04 |
| HV-ENT-002 | *Escherichia coli* | 5312420 | 197 | 125425 | 55 | 99.97 | 0.04 |
| HV-ENT-003 | *Klebsiella pneumoniae* | 5537652 | 121 | 191693 | 56 | 100 | 0.53 |

^1^ bp, base pairs.
